# Ticagrelor plasma concentration, metabolite ratio, and LDH level as independent risk factors for ticagrelor-induced dyspnea in ACS patients

**DOI:** 10.3389/fcvm.2026.1725673

**Published:** 2026-06-23

**Authors:** Yazi Zhao, Mengqi Gong, Yong Su, Jiaxin Zhao, Fangde Hong, Zheyuan Lu, Mengqing Ma, Meijie Jiang, Youfeng Liang

**Affiliations:** 1 Department of Pharmacy, the First Affiliated Hospital of Anhui Medical University, Hefei, China; 2 The Grade 3 Pharmaceutical Chemistry Laboratory of State Administration of Traditional Chinese Medicine,the First Affiliated Hospital of Anhui Medical University, Anhui Medical University, Hefei, China; 3 Department of Cardiology, the First Affiliated Hospital of Anhui Medical University, Hefei, China

**Keywords:** AR-C124910XX, lactate dehydrogenase, peak plasma concentration, ticagrelor, ticagrelor-induced dyspnea

## Abstract

**Introduction:**

Ticagrelor-induced dyspnea is a significant clinical concern and is associated with adverse outcomes. However, the specific risk factors leading to ticagrelor-induced dyspnea have not been fully elucidated. This study aims to identify risks by testing plasma concentrations of ticagrelor and its active metabolite, as well as other clinical factors that contribute to the risk.

**Methods:**

A total of 102 patients undergoing antiplatelet therapy (ticagrelor and aspirin) were enrolled, including 23 who developed ticagrelor-induced dyspnea and 79 who did not. Plasma levels of ticagrelor and its active metabolite, AR-C124910XX, were measured using high-performance liquid chromatography (HPLC). Clinical characteristics of all enrolled patients were collected completely. The influencing factors of ticagrelor-induced dyspnea were analyzed using logistic regression analysis, and the correlation between patient characteristics and plasma peak concentrations was examined using correlation analysis.

**Results:**

Compared with the non-dyspnea group, LDH levels and the ticagrelor metabolite ratio (AR-C124910XX/ticagrelor concentration ratio, TMR) decreased markedly in patients who developed dyspnea (LDH: *OR*=0.814 per 10 U/L, *P* = 0.027; TMR: *OR*=0.523 per 0.1 increase, *P* = 0.008). In contrast, peak ticagrelor concentration was markedly higher in the dyspnea group (*OR*=1.173 per 100 ng/mL, *P* = 0.012). The plasma peak concentration of ticagrelor was negatively related to LDH (*r* = −0.316, *P* = 0.001).

**Conclusion:**

The peak concentration of ticagrelor, plasma LDH level, and the TMR are important risk factors for associated with ticagrelor-induced dyspnea in ACS patients. The plasma concentration of ticagrelor is negatively correlated with the level of LDH.

## 1 Introduction

Dual-antiplatelet therapy (DAPT) with aspirin plus a P2Y12 receptor inhibitor is the principal treatment strategy for patients with acute coronary syndrome (ACS) or undergoing percutaneous coronary intervention (PCI) (1). Ticagrelor is a direct-acting, potent P2Y12 receptor inhibitor with a faster onset of action (peak activity within 30 min) and a shorter half-life (8–12 h) (2, 3). The drug blocks platelet aggregation by inhibiting the reversible binding of adenosine diphosphate (ADP), effectively preventing ischemic events (4). Ticagrelor can reduce the incidence of the composite of cardiovascular death, myocardial infarction, or stroke in patients with acute coronary syndromes (5–7). Current guidelines recommend ticagrelor as a first-line medication in antiplatelet therapy following ACS revascularization (8, 9). However, ticagrelor has been associated with higher rates of dyspnea; the incidence may vary depending on the clinical setting, and different studies have reported rates ranging from 6% to 20% (10–12). Ticagrelor-induced dyspnea can decrease adherence to therapy, potentially affecting treatment efficacy and recurrent cardiovascular events. Patients experiencing ticagrelor-induced dyspnea exhibited a significantly lower median duration of adherence to DAPT compared to those treated with clopidogrel (13). A meta-analysis suggested that the relative risk of dyspnea-related discontinuation was 6.4-fold higher for patients receiving ticagrelor than for those receiving a comparator and was associated with adverse clinical outcomes (14).

Recent studies have found variations in the pharmacokinetics of ticagrelor among different patients. The concentration of ticagrelor is directly related to platelet reactivity and the risk of adverse reactions, such as bleeding or dyspnea (15, 16). Ticagrelor is metabolized in the body by the hepatic cytochrome P450 (CYP) 3A and 3A5 enzymes, converting into its primary active metabolite, AR-C124910XX, which exhibits similar antiplatelet effects (17). Multiple factors influence the blood concentration of ticagrelor and AR-C124910XX. Studies have reported that patients with liver cirrhosis exhibit elevated ticagrelor concentrations and reduced AR-C124910XX concentrations (18). Women tend to have reduced excretion of ticagrelor and AR-C124910XX, leading to higher plasma concentrations (16). In elderly ACS patients, the peak concentration and area under the curve (AUC) of ticagrelor increase by approximately 25%, and the exposure of AR-C124910XX also increases (19). The study also suggested that ticagrelor-induced dyspnea was related to high ticagrelor concentration and low platelet aggregation (4, 16). Older age, male, history of asthma, stroke, or prior heart failure were reported to be possible predictors of ticagrelor-induced dyspnea (13). Other independent predictors included Asian race (lower risk), smoking, prior PCI or coronary artery bypass, hypercholesterolemia, peripheral artery disease, obesity, and older age (11). In light of limited evidence focusing on lower dose of ticagrelor, cardiologists increasingly emphasize individualized antithrombotic strategies (20, 21). This highlights the need for therapeutic drug monitoring of ticagrelor, and it is crucial to investigate the relationship between plasma levels of ticagrelor and its physiological effects in real-life patients undergoing treatment.

This study is based on the blood concentrations of ticagrelor and AR-C124910XX in ACS patients. It aims to analyze the correlation between blood concentrations and dyspnea, and explore the factors that influence it. The information was intended to provide a research basis for individualized medication with ticagrelor and aid in precise clinical drug administration.

## 2 Materials and methods

### 2.1 Study population

**Figure 1:**
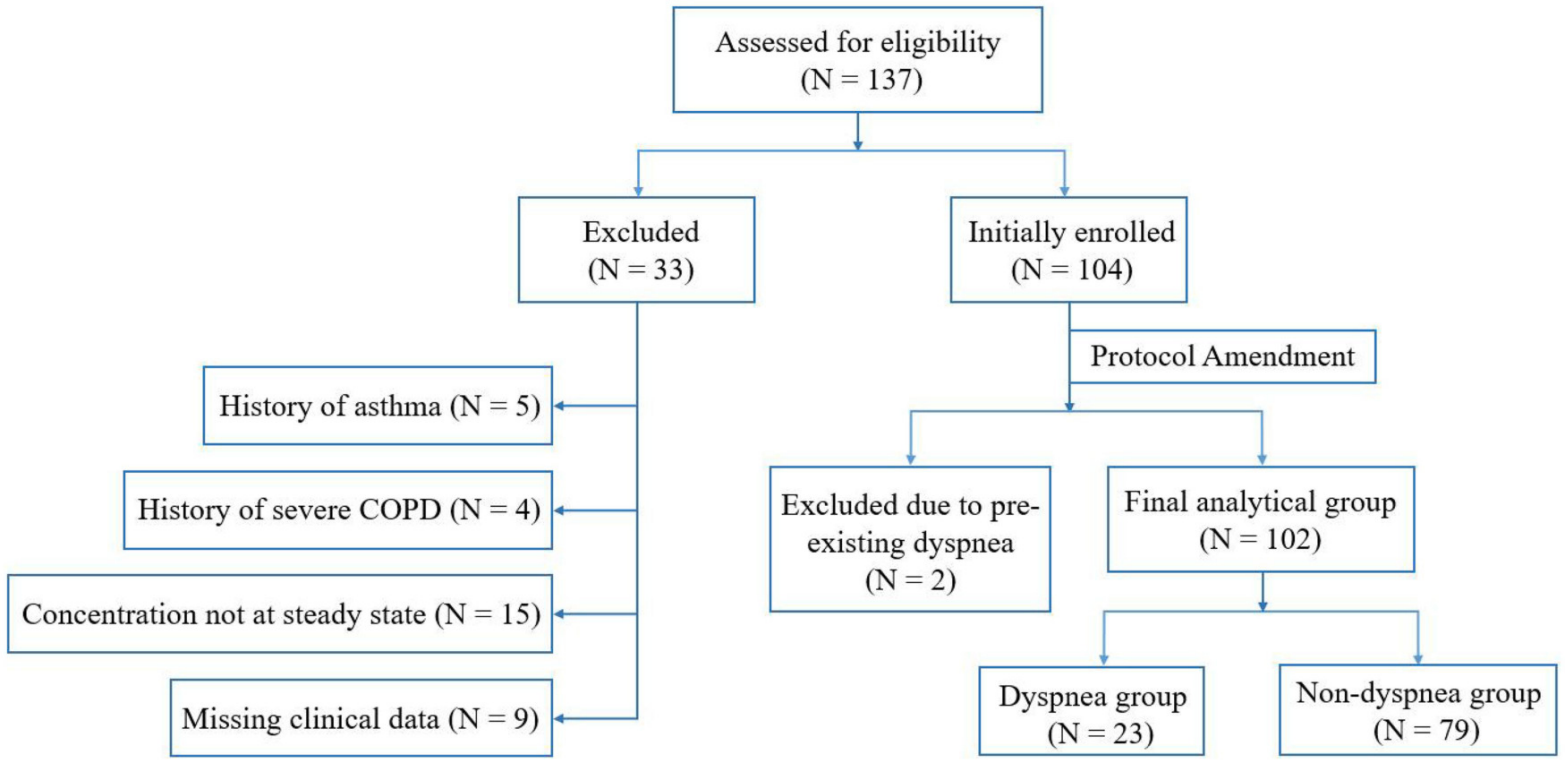
Participant flow diagram.

From August 2022 to December 2024, we recruited 102 inpatients hospitalized for ACS at the First Affiliated Hospital of Anhui Medical University, Hefei, China. All enrolled patients received standard dual antiplatelet therapy, consisting of ticagrelor (90 mg twice daily) and aspirin (100 mg once daily). Among the 102 patients, 23 developed ticagrelor-induced dyspnea, while 79 did not. The participant flow diagram is presented in Figure 1. In this study, suspected ticagrelor-induced dyspnea was characterized by *de novo* severe dyspnea after drug initiation, or worsening of frequency and severity in patients with pre-existing symptoms. Dyspnea was assessed using the modified Borg scale (22). In this study, the dyspnea symptoms were severe (modified Borg scale ≥ 5) and improved after discontinuation of ticagrelor or switching to an alternative antiplatelet agent. Specifically, symptom improvement was defined as a clinically meaningful reduction on the modified Borg scale (falling below scale ≤ 3) within 3 to 5 days of discontinuing ticagrelor or switching to an alternative P2Y12 inhibitor. This rapid clinical resolution aligns with the pharmacokinetic clearance time of ticagrelor, which strongly supports a drug-induced etiology.

Inclusion criteria were: inpatients diagnosed with ACS admitted to the First Affiliated Hospital of Anhui Medical University from August 2022 to December 2024; adults with an age of ≥ 18 years; accept antiplatelet drugs (ticagrelor and aspirin) or combination of antiplatelet drugs and anticoagulant (ticagrelor, aspirin, and rivaroxaban); Willing to comply with protocol-required blood sampling for drug concentration analysis. Furthermore, in our real-world clinical setting, individuals presenting with active systemic infections or severe anemia were excluded from the cohort, as these conditions are relative contraindications to coronary angiography and potent dual antiplatelet therapy.

Exclusion criteria were as follows: patients with a history of intracranial hemorrhage; those with a prior history of dyspnea before index admission; concomitant medication that might induce dyspnea; those with chronic obstructive pulmonary disease requiring continuous use of long-acting beta-agonists or long-acting muscarinic antagonists; those with history of asthma; The concentration of ticagrelor did not reach a stable state or the sampling time was unreasonable; or patients for whom clinical information such as weight and biochemical parameter could not be obtained. In the initial study design, we intended to capture both *de novo* dyspnea and acute exacerbations of pre-existing symptoms. However, to maximize diagnostic specificity and ensure the validity of the case definition, the protocol was refined during data collection. Ultimately, individuals with any documented history of dyspnea prior to the index admission were systematically excluded. Thus, the final analysis focused exclusively on *de novo* dyspnea occurring after ticagrelor initiation.

Informed consent was obtained from each patient, and the study protocol conformed to the ethical guidelines of the 1975 Declaration of Helsinki, as reflected in its prior approval by the institution's human research committee (Ethics Approval No: 2022131). All patients provided written informed consent before participation.

### 2.2 Determination of ticagrelor and AR-C124910XX plasma concentration

Blood samples were collected at least 60 h(five doses of ticagrelor)after ticagrelor therapy to ensure steady-state blood peak concentrations. Peripheral venous blood (2 mL) was collected in heparin tubes 2 h post-dose. The plasma was processed with methanol to precipitate proteins and then centrifuged at 12000g for 8 min. The collected supernatant was analyzed and validated using high-performance liquid chromatography (HPLC) in the hospital laboratory (23). The analyte was separated using acetonitrile and water (50:50) as a mobile phase on a Waters Alliance e-2695 model HPLC with an Agilent XB-C18 (4.6mm × 250 mm, 5 µm) column. HPLC parameters were set as follows: the detection wavelength was set at 299 nm, the flow rate was 1.0 mL/min, the column temperature was maintained at 30 °C, and the sample size was 20 μL. The retention times of ticagrelor and AR-C124910XX are 12.42 min and 11.84 min, respectively. The standard curve showed good linear relationships over 40–4,000 ng/mL for ticagrelor and 20–800 ng/mL for AR-C124910XX. For precision and accuracy, the intra- and inter-day precision (relative standard deviation, RSD) of 3 quality controls was <4.73%, and the accuracy ranged from 88.26% to 109.67%. The ticagrelor metabolite ratio (TMR) was calculated as the ratio of AR-C124910XX to ticagrelor blood concentration.

### 2.3 Collection of clinical data

The clinical data were extracted from the Hospital Information System. General information included sex, age, weight, and body mass index. Medical history comprised smoking status (categorized as current vs. previous or never), along with prior percutaneous coronary intervention (PCI) or percutaneous transluminal coronary angioplasty (PTCA). Laboratory parameters included complete blood count (white blood cell count, red blood cell count, hemoglobin, platelet count), liver function(alanine aminotransferase, aspartate aminotransferase, direct bilirubin, indirect bilirubin, lactate dehydrogenase), renal function (serum creatinine), and a heart failure biomarker (brain natriuretic peptide). All blood samples were routinely collected on the morning following hospital admission. Concomitant medication use was also documented, including statins (specifically atorvastatin vs. rosuvastatin), proton pump inhibitors (PPIs), CYP3A4 inhibitors (diltiazem, verapamil), and beta-blockers (metoprolol, bisoprolol).

### 2.4 Statistical analysis

All statistical analyses were performed using SPSS (version 22.0; IBM Corporation). The Kolmogorov–Smirnov test was used to analyze continuous variables. Continuous variables with a normal distribution were presented as mean ± standard deviation (SD) and compared using the independent samples t-test. For continuous variables that were not normally distributed, data were presented as median (interquartile range, IQR), and group comparisons were performed using the Mann–Whitney U test. Categorical variables were expressed as numbers (n) and percentages (%), and comparisons between groups were made using the ***χ*^2^** or Fisher's exact test. Multivariable logistic regression analysis was performed to identify factors associated with ticagrelor-induced dyspnea. Variables were selected based not solely on statistical significance (*P* < 0.05) but also on clinical relevance (age and body weight are known to be critical covariates of ticagrelor clearance). To avoid multicollinearity between ticagrelor concentration and TMR, these two highly correlated variables were not included in the same multivariable model. Instead, two separate parallel models were constructed: Model 1 included TMR and the covariates; Model 2 included ticagrelor concentration and the same covariates. This parallel approach ensured matrix stability and mitigated the risk of overfitting, given the limited number of dyspnea events. Correlations between clinical parameters and ticagrelor or AR-C124910XX plasma concentrations were analyzed using Pearson's correlation for normally distributed data or Spearman's rank correlation for non-normal data, respectively; A *p*-value < 0.05 was considered statistically significant. The predictive value of the identified factors was further assessed using receiver operating characteristic (ROC) curve analysis. The *P*-value reported for individual ROC curve tests the null hypothesis that the area under the curve (AUC) equals 0.5.The DeLong test was employed to compare ROC curves.

## 3 Results

### 3.1 Baseline characteristics

In this study, 102 patients were enrolled and categorized into two groups based on the presence or absence of ticagrelor-induced dyspnea. The age of the patients ranged from 33 to 80 years, and the majority were male (*n* = 76, 74.5%). All patients received conventional medical therapy, including beta-blockers, ACEI/ARB/ARNI (angiotensin-converting enzyme inhibitors/Angiotensin II receptor blockers/angiotensin receptor-neprilysin inhibitors), statins (atorvastatin or rosuvastatin), and antiplatelet agents. The baseline characteristics of the enrolled population are presented in Table 1.

**Table 1:** Characteristics of the patient population.

Characteristic		Dyspnea group (*n* = 23)	Non-dyspnea group (*n* = 79)	*χ*^2^/t/z	*P*
Demographic characteristics					
Male (%)	16 (69.6)		60 (75.9)	0.355	0.536
Age(years)	60.13 ± 11.39		59.18 ± 8.87	0.424	0.672
Weight(kg)	67.46 ± 12.86		70.34 ± 11.63	−1.020	0.310
BMI (kg/m^2^)	24.52 ± 3.3		24.95 ± 3.48	−0.533	0.595
Smoking (%)	7 (30.4)		21 (26.6)	0.127	0.716
Concomitant medications					
Statins	21 (91.3)		65 (82.3)	−0.003	0.978
PPIs (%)	18 (78.3)		62 (78.5)	0.001	0.982
CYP3A4 inhibitors (%)	1 (4.3)		2 (2.5)	0.163	0.65
*β*-blockers (%)	11 (47.8)		40 (50.6)	0.056	0.813
Concomitant diseases					
Prior PCI	8 (34.8)		19 (24.1)	0.954	0.305
Prior PTCA	2 (8.7)		4 (5.1)	0.340	0.515
Laboratory data					
WBC( × 10^9^/L)	6.14 (5.7, 8.0)		6.82 (5.4, 8.4)	0.092	0.927
RBC( × 10^9^/L)	4.43 ± 0.41		4.47 ± 0.6	−0.343	0.732
Platelets ( × 10^9^/L)	212 (170.3, 222.8)		195 (161.5, 226.0)	0.657	0.511
Hemoglobin (g/L)	135.52 ± 14.76		137.13 ± 18.28	−0.386	0.701
BNP (pg/mL)	27.48 (12.9, 81.6)		53.02 (19.8, 105.8)	−0.973	0.331
DBIL(*μ*mol/L)	2.88 (2.02, 3.4)		2.48 (1.7, 3.0)	1.506	0.132
IBIL(μmol/L)	10.89 (9.0, 16.0)		9.1 (7.3, 12.8)	1.866	0.062
ALT(U/L)	23.1 (15.8, 42.0)		25.8 (16.4, 41.9)	−0.156	0.876
AST(U/L)	24.5 (19.8, 37.0)		26.9 (20.2, 39.5)	−0.613	0.540
LDH(U/L)	158 (138.5, 170.1)		209.9 (172.1, 249)	−4.628	<0.001*
LDL-C(mmol/L)	2.34 ± 0.87		2.63 ± 0.94	−1.305	0.195
HDL-C(mmol/L)	1.18 ± 0.23		1.1 ± 0.25	1.413	0.161
Plasma concentration					
Ticagrelor C_max_(ng/mL)	697.6 (436.1, 1,072.2)		425.9 (228.4, 629.5)	3.543	<0.001*
ARC124910XX (ng/mL)	186.75 ± 120.02		182.7 ± 71.18	0.203	0.840
TMR	0.19 (0.15, 0.31)		0.41(0.28, 0.69)	−4.496	<0.001*

Values are number, number (%), mean ± standard deviation, or median (interquartile range).

The symbol * indicates a statistically significant difference with *p* < 0.05.

BMI, body mass index; PPI, Proton Pump Inhibitors; PCI, percutaneous coronary intervention; PTCA, percutaneous transluminal coronary angioplasty; WBC, white blood cell count; RBC, red blood cell count; BNP, brain natriuretic peptide; DBIL, direct bilirubin; IBIL, indirect bilirubin; ALT, alanine aminotransferase; AST, aspartate aminotransferase; LDH, lactate dehydrogenase; LDL-C, low-density lipoprotein cholesterol; HDL-C, high-density lipoprotein cholesterol; IQR, median with interquartile range; TMR, ticagrelor metabolite ratio (AR-C124910XX/ticagrelor concentration ratio).

Statins: Atorvastatin vs Rosuvastatin;.

CYP3A4 inhibitor: diltiazem, verapamil;.

β-blocker: metoprolol, bisoprolol.

### 3.2 Influence factors of ticagrelor-induced dyspnea

In the group of patients with ticagrelor-induced dyspnea, higher ticagrelor concentrations (*P* < 0.001) and lower LDH levels (*P* < 0.001) were observed, whereas AR-C124910XX concentration showed no statistically significant difference. A statistically significant trend toward a lower ticagrelor metabolite ratio (AR-C124910XX/Ticagrelor concentration ratio, TMR) was found in the dyspnea group (*P* < 0.001). Baseline levels of BNP, hemoglobin, and WBC counts were well balanced between the dyspnea and non-dyspnea groups, minimizing the likelihood that worsening heart failure, anemia, or infection confounded the assessment of ticagrelor-induced dyspnea. Details are shown in Table 1.

### 3.3 Correlations of ticagrelor and AR-C124910XX blood concentrations with clinical indicators

The monitoring results revealed substantial interindividual variability in the steady-state peak concentrations of ticagrelor among the 102 enrolled patients. Spearman's correlation analysis showed that ticagrelor concentrations were significantly correlated with LDH levels (Table 2). The concentrations of AR-C124910XX were positively correlated with age and negatively correlated with body weight and male (Tables 3, 4). These correlations were assessed using either Pearson's or Spearman's correlation test, depending on whether the data followed a normal distribution.

**Table 2:** Correlation analysis of ticagrelor blood concentration and other clinical indicators in patients with ACS.

Characteristic	*r*	*P*	Characteristic	*r*	*P*
Age(years)	0.027	0.786	DBIL(μmol/L)	0.119	0.232
Weight(kg)	−0.045	0.652	IBIL(μmol/L)	0.118	0.237
BMI (kg/m^2^)	0.004	0.969	ALT(U/L)	−0.026	0.799
WBC( × 10^9^/L)	−0.025	0.805	AST(U/L)	−0.093	0.354
RBC( × 10^9^/L)	−0.008	0.934	LDH(U/L)	−0.316	0.001*
Platelets ( × 10^9^/L)	−0.060	0.548	LDL-C(mmol/L)	−0.091	0.363
Hemoglobin (g/L)	−0.032	0.750	HDL-C(mmol/L)	−0.100	0.318
BNP (pg/mL)	−0.155	0.120	AR-C124910XX C_max_ (ng/mL)	0.379	<0.001*

The symbol * indicates a statistically significant difference with *p* < 0.05.

BMI, body mass index; WBC, white blood cell count; RBC, red blood cell count; BNP, brain natriuretic peptide; DBIL, direct bilirubin; IBIL, indirect bilirubin; ALT, alanine aminotransferase; AST, aspartate aminotransferase; LDH, lactate dehydrogenase; LDL-C, low-density lipoprotein cholesterol; HDL-C, high-density lipoprotein cholesterol; IQR, median with interquartile range.

**Table 3:** Correlation analysis of AR-C124910XX blood concentration and other clinical indicators in patients with ACS.

Characteristic	*t/r*	*P*	Characteristic	*t/r*	*P*
Age(years)	0.249	0.012*	DBIL(μmol/L)	0.064	0.524
Weight(kg)	−0.294	0.003*	IBIL(μmol/L)	0.129	0.195
BMI (kg/m2)	−0.112	0.263	ALT(U/L)	0.130	0.193
WBC( × 10^9^/L)	−0.075	0.451	AST(U/L)	0.047	0.641
RBC( × 10^9^/L)	−0.193	0.051	LDH(U/L)	0.015	0.878
Platelets ( × 10^9^/L)	−0.111	0.268	LDL-C(mmol/L)	0.125	0.210
Hemoglobin (g/L)	−0.182	0.067	HDL-C(mmol/L)	0.045	0.656
BNP (pg/mL)	0.014	0.889	Ticagrelor C_max_ (ng/mL)	0.421	<0.001*

The symbol * indicates a statistically significant difference with *p* < 0.05.

BMI, body mass index; PCI, percutaneous coronary intervention; PTCA, percutaneous transluminal coronary angioplasty; WBC, white blood cell count; RBC, red blood cell count; BNP, brain natriuretic peptide; DBIL, direct bilirubin; IBIL, indirect bilirubin; ALT, alanine aminotransferase; AST, aspartate aminotransferase; LDH, lactate dehydrogenase; LDL-C, low-density lipoprotein cholesterol; HDL-C, high-density lipoprotein cholesterol; IQR, median with interquartile range.

**Table 4:** Influence of sex, smoking, statins, prior PCI, and prior PTCA on ticagrelor and AR-C124910XX concentration.

	Ticagrelor concentration			AR-C124910XX concentration
	*z*	*P*	*t*	*P*
Male (%)	−0.303	0.762	−4.127	<0.001*
Smoking (%)	0.836	0.403	0.659	0.512
Statins	−0.028	0.978	1.658	0.100
Prior PCI	1.699	0.089	−1.002	0.319
Prior PTCA	−0.967	0.333	−1.340	0.183

The binary categorical variables of gender, smoking, statins, history of PCI, and PTCA were tested by the T-test and the U-test.

The symbol * indicates a statistically significant difference with *p* < 0.05.

PCI, percutaneous coronary intervention; PTCA, percutaneous transluminal coronary angioplasty; Statins: Atorvastatin vs Rosuvastatin.

**Table 5:** Logistic regression analysis of risk factors for ticagrelor-induced dyspnea.

Factor	OR	95%CI	*P*
Age	1.016	0.951–1.085	0.632
Weight	0.983	0.929–1.040	0.546
LDH	0.814	0.681–0.974	0.027
TMR	0.523	0.323–0.846	0.008

To enhance clinical interpretability, continuous variables were scaled to clinically meaningful increments: TMR per 0.1 increase, LDH per 10 U/L, and peak ticagrelor concentration per 100 ng/mL. Odds ratios (ORs) and 95% confidence intervals (CIs) are presented based on these scaled units.

LDH, lactate dehydrogenase; TMR, Ticagrelor metabolite ratio (AR-C124910XX/ticagrelor concentration ratio).

### 3.4 Multivariable predictors and predictive performance for ticagrelor-induced dyspnea

In binary logistic regression analysis, TMR (*OR*=0.523, 95% *CI*: 0.323–0.846, *P* = 0.008) and LDH (*OR*=0.814, 95% *CI:* 0.681–0.974, *P* = 0.027) were significantly associated with ticagrelor-induced dyspnea (Table 5). To enhance clinical interpretability, these continuous variables were scaled to clinically meaningful increments (TMR per 0.1 increase, LDH per 10 U/L increase, and peak ticagrelor concentration per 100 ng/mL increase). After adjusting for age and weight, higher TMR and higher LDH levels remained associated with a lower likelihood of dyspnea. In a separate parallel multivariable model evaluating the parent drug, peak ticagrelor concentration (scaled per 100 ng/mL) was independently associated with dyspnea (*OR*=1.173, 95% *CI*: 1.036–1.327, *P* = 0.012) after adjusting for age, weight, and LDH. Collectively, peak ticagrelor concentration, plasma LDH level, and TMR are independently associated with ticagrelor-induced dyspnea in patients with ACS.

The metabolic ratio (TMR) was further analyzed by dividing it into four quartile groups (< 0.239, 0.239–0.339, 0.339–0.552, and > 0.552). The chi-square test suggested a significant association between the metabolic ratio and the incidence of ticagrelor-induced dyspnea (*χ*^2^ = 18.754, *P* < 0.001), indicating that patients with a metabolic ratio exceeding 0.552 were at low risk of dyspnea (Table 6).

**Table 6:** Correlation between metabolic ratios and ticagrelor-induced dyspnea in patients.

Metabolic ratio	Dyspnea group (*n* = 23)	Non-dyspnea group (*n* = 79)	χ^2^	*P*
Q1 (<0.239)	13	13	18.754	<0.001
Q2 (0.239∼0.339)	5	20		
Q3 (0.339∼0.552)	5	21		
Q4(>0.552)	0	25		

**Figure 2:**
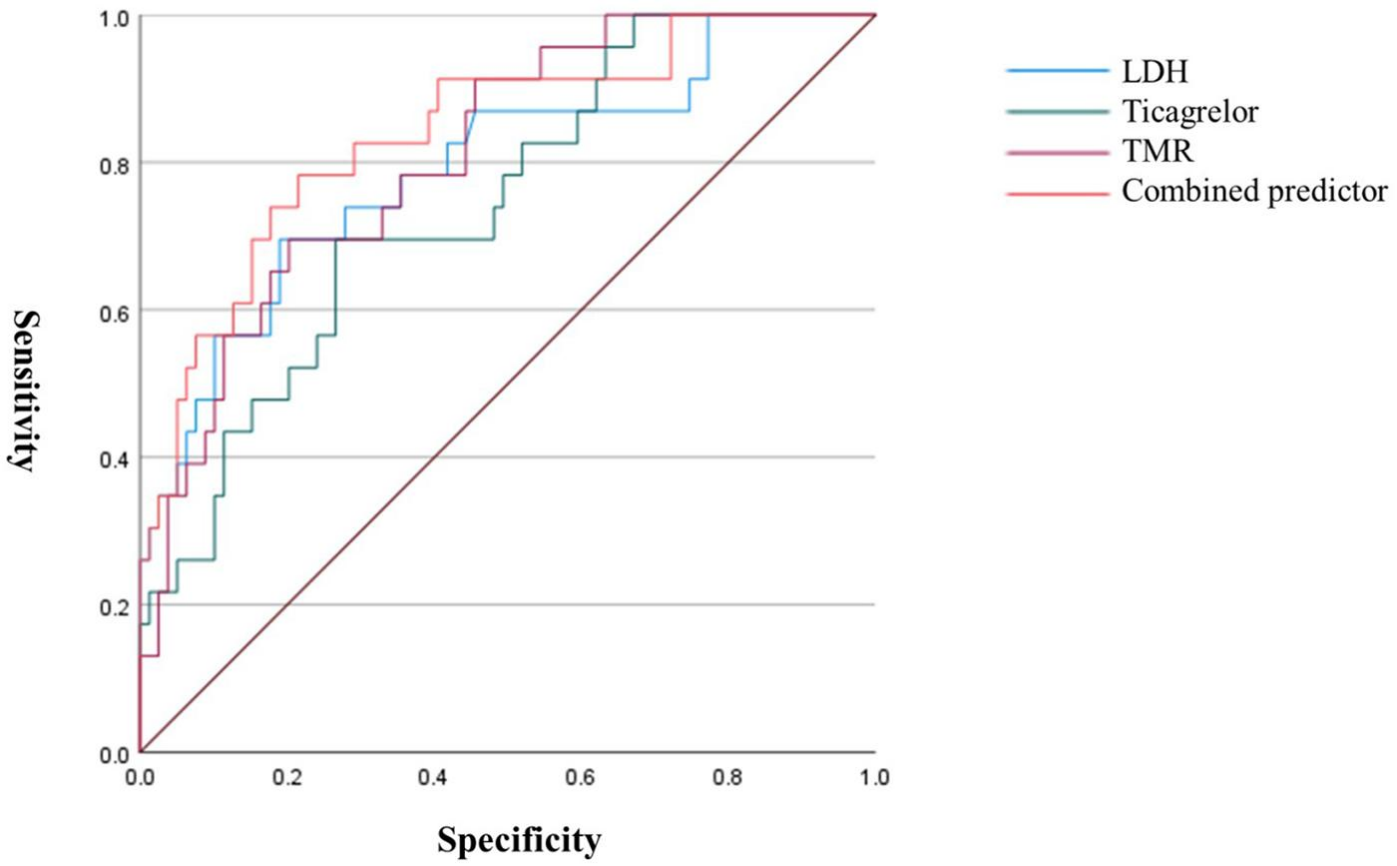
ROC curve analysis of risk factors for the prediction of ticagrelor dyspnea.

Finally, receiver operating characteristic (ROC) curve analysis confirmed the predictive value of these indicators. The AUC for ticagrelor concentration was 0.744 (95% *CI*: 0.634–0.853, *P* < 0.001), while TMR achieved an AUC of 0.809 (95% *CI*: 0.715–0.903, *P* < 0.001). Notably, the combined predictor of LDH and TMR achieved the highest AUC of 0.840 (95% *CI*: 0.745–0.935, *P* < 0.001), with a maximum Youden index of 0.567. The DeLong test was used to evaluate the additive value of LDH. Results showed that adding LDH did not significantly improve the AUC compared to TMR alone (*P* = 0.592).These findings indicate that ticagrelor plasma concentration and TMR possess significant predictive value for ticagrelor-induced dyspnea (Figure 2).

## 4 Discussion

This is the first systematic study to evaluate the correlation between the steady-state peak concentration (Cmax) of ticagrelor and its metabolic ratio, as well as the adverse reaction of dyspnea, in a real-world clinical setting. Compared to previous clinical studies focusing on trough concentration (15, 24), this study innovatively revealed the clinical significance of ticagrelor Cmax and TMR. The study results showed that, unlike trough concentrations, which are affected by factors such as age and sex (25, 26), Cmax were not influenced by these factors. This discrepancy is likely attributable to the fact that peak concentrations are primarily determined by post-dose drug absorption, whereas trough concentrations are influenced by both absorption and systemic drug clearance. Since drug clearance rates are generally associated with age, sex, and body weight, the degree of interindividual variability in peak concentrations is smaller than that in trough concentrations across individuals of different ages, sexes, and body weights. Conversely, the blood concentration of the active metabolite shows a significant correlation with age, sex, and body weight, which were consistent with previous literature reports (16, 19, 24).

Another key finding is that TMR has good predictive value in patients with dyspnea associated with ticagrelor. In this study, a TMR > 0.552 (indicating relatively rapid ticagrelor metabolism and a low proportion of the parent drug) was associated with a significantly reduced risk of ticagrelor-associated dyspnea. This aligns with Tamakauskas et al. (27), who reported that lower ADP-induced platelet aggregation (reflecting stronger P2Y12 inhibition) indicated elevated plasma concentrations and enhanced ticagrelor activity which might relate to dyspnea. Thus, a reduced TMR, reflecting slower parent drug metabolism, is a key metabolic characteristic associated with an increased risk of dyspnea which was confirmed in this study. To assess this predictive value and to develop more individualized treatment strategies, this study conducted ROC curve analysis with the aim of maximizing the cardiovascular protective effect of ticagrelor while minimizing the associated risk of dyspnea. Although the AUC value of this model reached 0.840(95% CI: 0.745–0.935), indicating good overall discriminatory performance, the maximum Youden index was 0.567 (sensitivity 0.783, specificity 0.785), suggesting room for improvement in balancing sensitivity and specificity. This may be related to the fact that the proportion of negative samples was as high as 77.5%; the model tends to increase specificity, thereby limiting further improvement of the Youden index. Additional optimization can be achieved by increasing the sample size or introducing cost-sensitive learning strategies in the future. Currently, this model already has practical value as an auxiliary tool for exclusion diagnosis.

**Figure 3:**
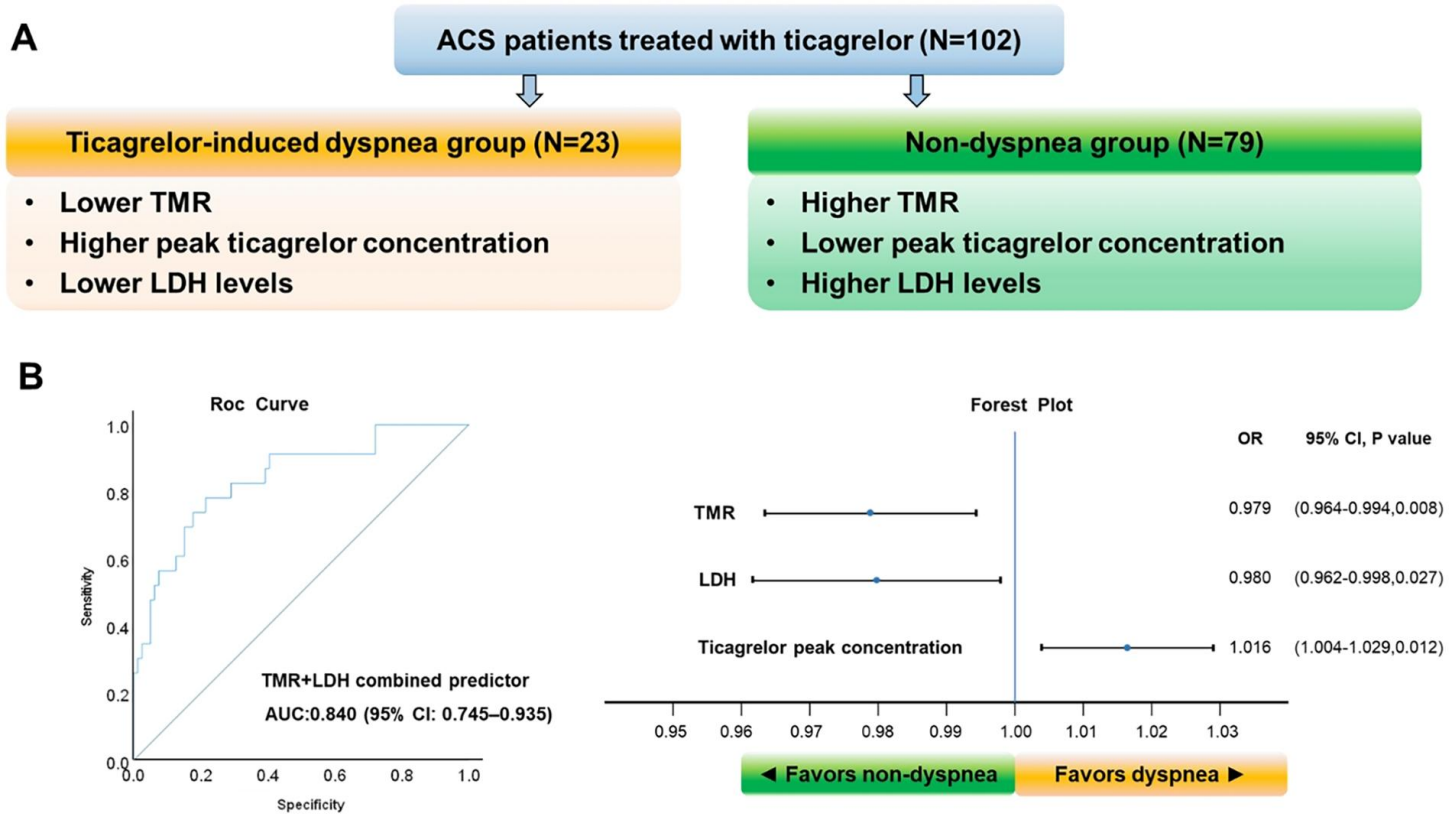
Central illustration. Factors associated with ticagrelor-induced dyspnea in patients with acute coronary syndrome. **(A)** Clinical, pharmacokinetic, and biochemical characteristics stratified by the presence of ticagrelor-related dyspnea. **(B)** Left: ROC curve for the model predicting ticagrelor-induced dyspnea. Right: Forest plot of multivariable logistic regression (continuous variables scaled to TMR per 0.1, LDH per 10 U/L, and peak concentration per 100 ng/mL). Low TMR, low LDH levels, and high peak ticagrelor concentration were associated with a higher probability of dyspnea. ACS, acute coronary syndrome; TMR, Ticagrelor metabolite ratio (AR-C124910XX/ticagrelor concentration ratio); LDH, lactate dehydrogenase.

Our pharmacokinetic-pharmacodynamic analysis revealed a negative correlation between LDH levels and ticagrelor concentrations, as well as significantly lower LDH levels in the dyspnea group. Previous studies have suggested that ticagrelor, beyond its antiplatelet effects, may attenuate tissue damage and inflammatory cascades through multiple mechanisms (28–32) and also exert protective effects on other organs (29, 33, 34). LDH is a classic marker of ischemic injury, a reduction in LDH levels could reflect decreased tissue injury. One possible interpretation, therefore, is that ticagrelor, by alleviating myocardial ischemia, may reduce LDH release. Furthermore, the DeLong test revealed that adding LDH to TMR did not provide significant additive predictive value. This finding supports our hypothesis that, rather than being a mechanistic driver of dyspnea, lower LDH levels may simply reflect that individuals with higher ticagrelor exposure experience greater anti-inflammatory or tissue-protective benefits while also being more susceptible to concentration-dependent adverse effects such as dyspnea. Moreover, given the observational nature of our study, the absence of inflammatory biomarkers (e.g., neutrophils, C-reactive protein), and the modest sample size that limited our multivariable analysis, the hypothesis that LDH levels reflect the anti-inflammatory or tissue-protective effects of ticagrelor remains speculative. These findings remain correlational, and further investigations with larger cohorts and comprehensive inflammatory profiling are necessary to clarify this relationship. In addtion, alternative explanations should also be considered, such as baseline differences in organ function, comorbidities, or unmeasured confounders. The key associations among TMR, peak plasma concentration, LDH levels, and the risk of ticagrelor-related dyspnea are visually summarized in the Central Illustration (Figure 3).

This study had several limitations. First, the current investigation was constrained by a modest sample size, attributable to the strict inclusion criteria. In clinical practice, several patients developed dyspnea symptoms after the first or second dose discontinued the drug early and were excluded. Consequently, our findings primarily reflect the pharmacokinetics of late-onset, steady-state dyspnea. However, TMR is a relative measure of metabolic efficiency. Unlike peak concentrations, which are highly susceptible to sampling timing, TMR reflects an individual's inherent metabolic capacity. Therefore, although the absolute concentration thresholds we reported may be specific to steady-state conditions, the TMR-based findings are likely more generalizable and may still apply to early dyspnea. Second, to ensure cohort homogeneity and avoid confounding by baseline cardiopulmonary conditions, the protocol was amended to focus exclusively on *de novo* dyspnea. The protocol amendment, which excluded individuals with a prior history of dyspnea, limits the generalizability of our findings to that specific subpopulation and may introduce selection bias. Our findings predominantly apply to *de novo* ticagrelor-induced dyspnea. Third, the negative correlation between LDH levels and peak ticagrelor concentrations was identified through *post hoc* analysis. The absence of serial LDH assessments limits to analyze dynamic changes in LDH during treatment. Finally, the diagnosis of ticagrelor-induced dyspnea in our cohort was primarily based on clinical dechallenge (symptom improvement following drug cessation). Because a formal re-challenge protocol was clinically contraindicated, this rapid improvement strongly supports, but cannot definitively establish, absolute causality. Moving forward, we plan to incorporate the polymorphism analysis of key genes such as ABCB1 and UGT1A1, and establish a prediction model based on the concentration-effect relationship.

## 5 Conclusion

This study suggests that a higher peak ticagrelor concentration, a low metabolic ratio (TMR), and lower LDH levels are associated with ticagrelor-induced dyspnea in patients with ACS. These findings suggest that biochemical profiling and therapeutic drug monitoring may help identify high-risk individuals, thereby facilitating individualized antiplatelet strategies.

## Data Availability

The raw data supporting the conclusions of this article will be made available by the authors, without undue reservation.
